# Supplementary material for: Conditional chemoconnectomics (cCCTomics) as a strategy for efficient and conditional targeting of chemical transmission
Source: eLife. 2024 Apr 30;12:RP91927. doi: 10.7554/eLife.91927 (PMC11060718; doi:10.7554/eLife.91927)
Supplement: Figure 3—source data 3. [file elife-91927-fig3-data3.zip › Figure 3 source data3 part 1.zip!/Figure 3-Source data 3-part1/1071f2-1-CRISPResso_Report_ztwktvfp.zip!/CRISPResso_on_1071f2_3/1a.Read_barplot.pdf]

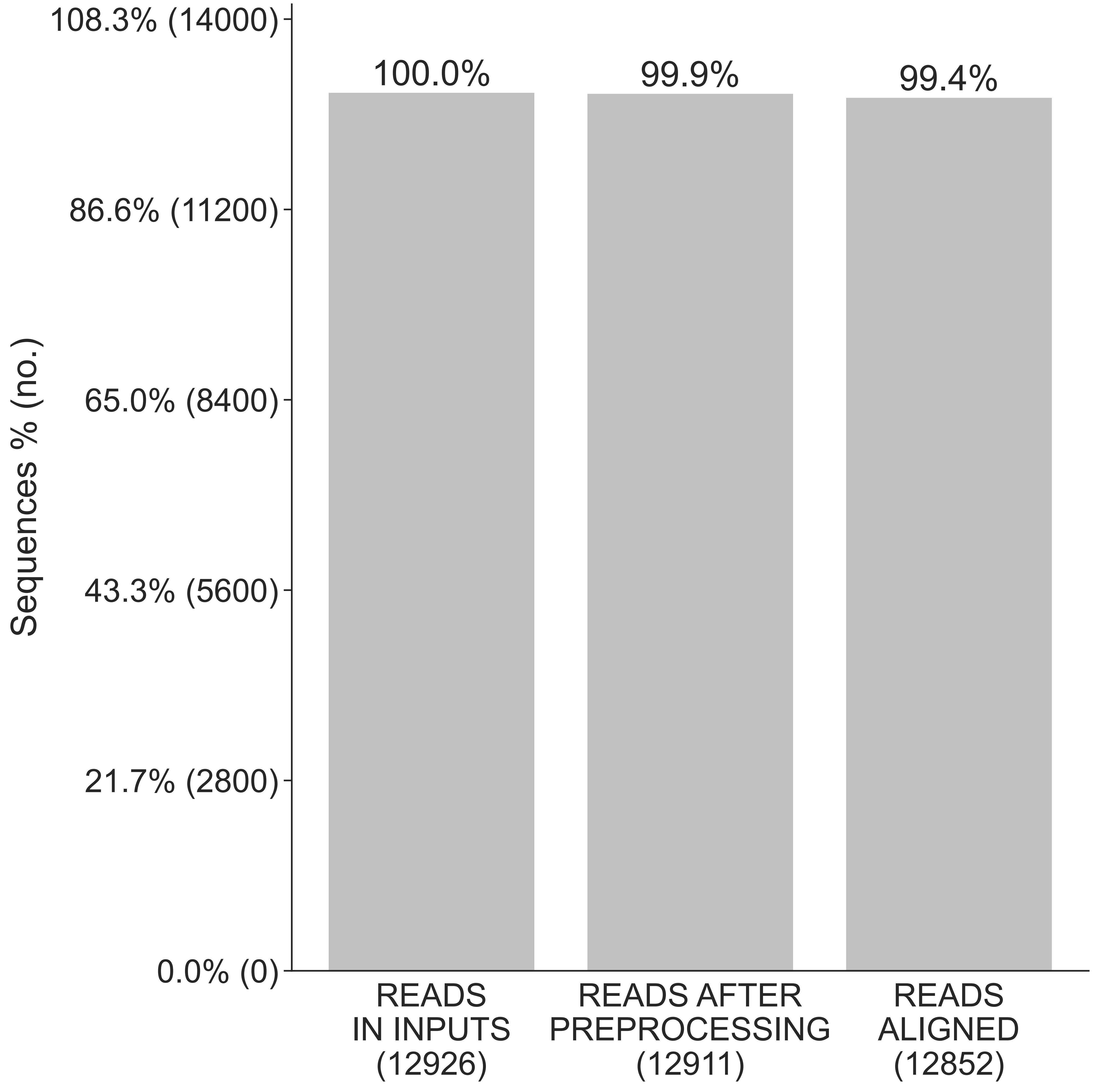
Sequences % (no.)
108.3% (14000)
86.6% (11200)
65.0% (8400)
43.3% (5600)
21.7% (2800)
0.0% (0)
100.0%
99.9%
99.4%
READS
IN INPUTS
(12926)
READS AFTER
PREPROCESSING
(12911)
READS
ALIGNED
(12852)
